# The Relationship Between Negative Life Events and Aggressive/Prosocial Behavior: A Latent Profile Analysis Based on Basic Psychological Need Satisfaction

**DOI:** 10.3390/bs15121722

**Published:** 2025-12-12

**Authors:** Yanli Zhu, Mengzhu Jiang, Fan Feng, Defeng Xia

**Affiliations:** 1 Department of Psychology, School of Education, Zhengzhou University, Zhengzhou 450001, China; 2 School of Political Science and Public Administration, Zhengzhou University, Zhengzhou 450001, China

**Keywords:** negative life events, prosocial behavior, aggressive behavior, latent profile analysis, communal orientation

## Abstract

This study integrates Self-Determination Theory with an emotion–cognition framework and uses a questionnaire-based design to examine individual differences in behavioral responses. A person-centered latent profile analysis (LPA) was conducted among 1596 Chinese participants (gender: 68.9% female; mean age: 18.25 years old) to identify distinct behavioral profiles in the context of negative life events. The results revealed that (1) four distinct profiles emerged among participants: a Self-Focused Group (33.65%), a Stress–Aggression Group (20.11%), an Ambivalent–Aggression Group (21.68%), and a Prosocial Group (24.56%); (2) significant differences were observed among these profiles in terms of empathic responses and psychological need satisfaction. By employing a person-centered analytical approach, this study contributes to a nuanced understanding of behavioral divergence under stress and offers insights for designing subgroup-specific psychological interventions.

## 1. Introduction

Negative life events refer to stressful situations that elicit adverse emotional experiences in individuals, such as interpersonal conflicts and academic pressures. The behavioral outcomes of these events remain a subject of debate. General Strain Theory (Agnew, 1992) posits that individuals may exhibit aggressive behaviors in response to environmental stressors as a means of venting negative emotions. In contrast, the Transformative Theory of Trauma (Vollhardt, 2009; Zaki, 2014) suggests that individuals may develop heightened empathy following traumatic experiences, thereby increasing their likelihood of engaging in prosocial behaviors. Emerging adulthood (ages 18–25) is a developmental period marked by heightened exposure to academic and interpersonal stress, which can strongly influence emotional regulation and social behavior (Arnett, 2000; Zimmer-Gembeck & Skinner, 2016). As the satisfaction of basic psychological needs is crucial for adaptive functioning during this stage (Vansteenkiste et al., 2020), exploring how negative life events and need satisfaction jointly shape behavioral responses offers insight into individual differences in stress adaptation. The question of why individuals respond differently to similar negative life events remains unresolved, and the underlying motivational mechanisms merit further exploration.

Self-Determination Theory (Deci & Ryan, 2000) underscores the importance of fulfilling three fundamental psychological needs: autonomy, competence, and relatedness as essential for the emergence and sustenance of intrinsic motivation. Motivation is not derived from a single source but rather from the interaction of cognitive, emotional, and contextual factors. Current research presents two primary perspectives on the origins of prosocial motivation. The first perspective emphasizes the significance of external contexts in eliciting behavioral tendencies through mechanisms such as emotional responses or cognitive appraisals triggered by situational cues. This “emotion–cognition-driven” viewpoint encompasses processes like empathic responses and cost–benefit evaluations (Meglino & Korsgaard, 2006). The second perspective centers on the fulfillment of internal needs, positing that individuals engage in helping behaviors based on value identification and autonomous choice—termed “autonomous motivation” (Böckler et al., 2016). Although these perspectives are theoretically distinct, they are not mutually exclusive in practice. For example, emotional experiences such as empathy may foster a sense of relatedness that further reinforces internalized values while facilitating a transition from reactive to proactive helping behaviors. This process illustrates the dynamic nature of motivation shaped by interactions between individual characteristics and contextual influences.

Accordingly, this study employs Self-Determination Theory as the primary theoretical framework to investigate how the fulfillment of basic psychological needs influences individuals’ tendencies toward social behavior—specifically, prosocial versus aggressive behaviors—in the context of negative life events. To enhance our understanding of how these needs manifest in social behavior, the study incorporates situational expressions of motivation by selecting three relevant psychological constructs as proxies for the three fundamental needs.

Specifically, “communal orientation” is conceptualized as the behavioral manifestation of the need for autonomy. It underscores voluntary assistance driven by intrinsic values rather than social pressure or external rewards. Prior research has demonstrated that communal orientation accentuates the non-instrumental and self-determined nature of helping behavior (Clark & Mils, 1993; Guo et al., 2021), thereby serving as a natural extension of autonomy needs within prosocial orientations. When individuals perceive caring for others as integral to their self-chosen values, their motivation becomes more internalized, leading to more sustainable prosocial behaviors (Weinstein & Ryan, 2010). Empathy, widely acknowledged as a fundamental driver of prosocial behavior, serves to represent the psychological reflection of the need for relatedness. Meta-analytic findings reveal a significant positive correlation between empathy and prosocial behavior, a relationship that remains stable across diverse cultures, age groups, and contexts (Yin & Wang, 2022). Empathy encompasses four dimensions: perspective-taking, imaginative transposition, empathic concern, and personal distress. High levels of empathy enable individuals to cultivate close relationships and social connections, thus fulfilling their need for relatedness (Saulin et al., 2024). Notably, the dimensions of empathic concern and perspective-taking are strongly linked with relationship satisfaction and social integration, constituting essential psychological foundations for belongingness (Batson, 2009). Finally, “prosocial self-efficacy” is utilized to reflect the need for competence. This construct encapsulates individuals’ beliefs regarding their capacity to effectively engage in helping behaviors (Cuadrado et al., 2016).

Unlike traditional questionnaire-based studies that primarily rely on variable-centered analyses, latent profile analysis (LPA) transcends the mere examination of correlations or causal relationships among variables. Instead, it identifies naturally occurring subgroups within a sample based on patterns across various motivational dimensions. This person-centered approach not only uncovers individual differences within the sample but also reveals distinct behavioral patterns associated with different motivational profiles. Thus, it offers more detailed and nuanced explanations compared with traditional methodologies (Pan et al., 2021). Consequently, LPA is particularly well-suited for elucidating the diversity of individual motivations in response to negative life events. It also helps clarify the behavioral implications of these motivational profiles, providing an integrative perspective for addressing existing theoretical debates.

In this study, latent profile analysis was used to incorporate negative life events, motivation, situation-relevant psychological constructs, and behavioral tendencies as classification indicators. The aim is to identify distinct behavioral response patterns among individuals. It also explores how motivational differences contribute to their divergent tendencies toward prosocial or aggressive behavior (Howard & Hoffman, 2018).

## 2. Method

### 2.1. Participants

Using a stratified sampling method, both online and offline questionnaire surveys were conducted across seven schools of varying tiers in a central province of China, including key universities, general universities, and vocational/private institutions. A total of 1596 valid responses were collected. Among the participants, 491 were male (30.8%), 1099 were female (68.9%), and gender information was missing for 6 participants. The average age was 18.75 years (SD = 1.04). Approximately 24.9% of the participants reported having experienced being “left-behind children,” while 74.8% had no such experience; data were missing for 3 participants. All participants were informed of the study purpose and gave their consent prior to participation.

To examine the pattern of missing data, Little’s MCAR test was conducted, yielding a non-significant result (*p* = 0.25). Therefore, the null hypothesis was accepted, indicating that the missing data could be treated as missing completely at random (MCAR). Given that the overall proportion of missing data points was below 20%, multiple imputation was employed under the MCAR assumption, with five imputed datasets generated and pooled to obtain unbiased parameter estimates for subsequent analyses.

### 2.2. Measures

#### 2.2.1. Negative Life Events Scale

The Adolescent Self-Rating Life Events Checklist (Chen et al., 2016) was used to assess the number of negative life events experienced by participants, the overall stress level, and their sensitivity to these stressors over the past 12 months. The scale includes 27 items representing negative life events, covering five core dimensions: punishment (e.g., physical punishment or severe scolding by parents), loss (e.g., death of a family member or close friend), interpersonal stress (e.g., conflict with classmates or close friends), academic pressure (e.g., high academic workload), and adaptation problems (e.g., long-term separation from family without reunion). An additional category of “other events” is included as a supplementary dimension. Items are rated on a 6-point Likert scale. The internal consistency reliability (Cronbach’s α) of the total scale was 0.96. The Cronbach’s α coefficients for each subscale were as follows: interpersonal stress (0.86), academic pressure (0.80), punishment (0.94), loss (0.86), adaptation problems (0.73), and other events (0.77).

#### 2.2.2. Prosocial Behavior Scale

The Prosocial Tendencies Measure (Kou et al., 2007) was used to assess prosocial behavior. It comprises six subscales: public (e.g., I try my best to help others when people are watching), compliant (e.g., I rarely refuse when others ask me for help), altruistic (e.g., I help others not for the expectation of reciprocal rewards in the future), anonymous (e.g., I prefer to donate anonymously), dire (e.g., I tend to help those who are truly in urgent need), and emotional prosocial behaviors (e.g., I often help others when they are in a very low mood), with a total of 26 items. Responses are rated on a 5-point Likert scale, with higher scores indicating a greater tendency toward prosocial behavior. In the present study, the internal consistency of the total scale was high (Cronbach’s α = 0.95).

#### 2.2.3. Aggressive Behavior Scale

The Reactive–Proactive Aggression Questionnaire (W. L. Zhang et al., 2014) was used to assess aggressive tendencies. The scale consists of 20 items, divided into two subscales: reactive aggression (e.g., I shout at someone when they make me angry) and proactive aggression (e.g., I hurt others to win a competition). Responses are rated on a 6-point Likert scale, with higher scores indicating stronger aggression tendencies. In this study, the internal consistency of the total scale was Cronbach’s α = 0.92.

#### 2.2.4. Basic Psychological Needs Satisfaction Scale

The Chinese version of the Basic Psychological Need Satisfaction Scale (Liu et al., 2013) was employed to measure the extent to which participants’ basic psychological needs were met. It consists of 21 items across three dimensions: autonomy (e.g., I feel that I can decide for myself how to live), competence (e.g., I have recently had the ability to learn new knowledge or skills), and relatedness (e.g., People in my life care about me), rated on a 7-point Likert scale. Higher scores reflect greater satisfaction of psychological needs. Reliability testing showed that the overall internal consistency was Cronbach’s α = 0.91; the subscale reliabilities were 0.72 for autonomy, 0.78 for competence, and 0.91 for relatedness.

#### 2.2.5. Interpersonal Reactivity Index

The Chinese version of the Interpersonal Reactivity Index (F. F. Zhang et al., 2010) was used to measure empathy. The scale contains four subscales: perspective taking (e.g., Before making decisions, I try to see each person’s point of view), fantasy (e.g., I do immerse myself in the emotional world of the characters), empathic concern (e.g., I am a fairly soft-hearted person), and personal distress (e.g., In emergency situations I feel worried, afraid, and have difficulty calming down), with a total of 22 items. Responses are rated on a 5-point Likert scale, with higher scores indicating greater empathic ability. In this study, the internal consistency reliability of the total scale was Cronbach’s α = 0.76; subscale reliabilities were as follows: perspective taking (0.74), fantasy (0.63), empathic concern (0.64), and personal distress (0.76).

#### 2.2.6. Communal Orientation Scale

The Communal Orientation subscale of the Interpersonal Relationship Orientation Scale (Wang & Li, 2015) was used to assess the extent to which individuals prioritize others’ needs in social interactions. The scale includes 14 items rated on a 5-point Likert scale, with higher scores indicating a stronger communal orientation (e.g., When making a decision, I take other people’s needs and feelings into account). In this study, the internal consistency coefficient was Cronbach’s α = 0.71.

#### 2.2.7. Prosocial Self-Efficacy Scale

The Prosocial Self-Efficacy Scale was originally developed by Cuadrado et al. (Cuadrado et al., 2016), consisting of 5 items on a single dimension, rated on a 7-point Likert scale. Higher scores indicate greater perceived prosocial self-efficacy (e.g., I can adopt behavior oriented to help others). The scale was adapted into Chinese following Brislin’s translation framework (Sousa & Rojjanasrirat, 2010). Specifically, two professionals independently completed the initial translation. After discussion and comparison, a preliminary version was developed. A native English-speaking expert in education then performed a back-translation. Finally, psychology researchers and translation experts jointly reviewed and refined the back-translated version by comparing it with the original to produce the final Chinese version.

Psychometric analysis showed that the internal consistency of the scale was Cronbach’s α = 0.91. The Kaiser–Meyer–Olkin (KMO) value was 0.88, and Bartlett’s test of sphericity was significant (*p* < 0.001), indicating suitability for factor analysis. Exploratory factor analysis supported a single-factor structure, explaining 73.72% of the total variance. Confirmatory factor analysis conducted using R showed good model fit: CFI = 0.97, TLI = 0.95, SRMR = 0.03, RMR = 0.03, and RMSEA = 0.13. Although the RMSEA was relatively high, the other indices (CFI, TLI, SRMR, and RMR) indicated excellent fit. This discrepancy may be due to the sensitivity of RMSEA in small, single-factor models (SEM, n.d.); SRMR is considered more appropriate for evaluating such simple models (Hu & Bentler, 1999). Taken together with theoretical considerations and the performance of other indices, the model was deemed acceptable.

### 2.3. Statistical Analysis

The study first conducted correlation analyses among 17 measured indicators to examine relationships between variables. Subsequently, standardized scores of these indicators were used as manifest variables to construct a latent profile model. Aggressive and prosocial behaviors, along with the other psychological indicators, were included in the latent profile model in order to identify naturally occurring patterns of co-occurring behaviors and motivational states among participants. Model fit was evaluated to identify subgroups of participants with similar profiles in terms of prosocial behavior, aggressive behavior, basic psychological needs, empathy, communal orientation, prosocial self-efficacy, and negative life events.

Latent profile analysis (LPA) is a person-centered statistical approach used to classify individuals into homogeneous subgroups (profiles) based on their responses. The classification results estimate the probability that an individual belongs to a particular latent profile. To determine the optimal number of profiles and reduce the potential influence of estimation errors, six model fit indices were used in this study: Akaike Information Criterion (AIC), Bayesian Information Criterion (BIC), adjusted BIC (aBIC), Lo-Mendell-Rubin adjusted likelihood ratio test (LMRT), bootstrap likelihood ratio test (BLRT), and entropy. A model with lower AIC, BIC, and aBIC values, higher entropy, and significant p-values for LMRT and BLRT is considered to have better model fit. Entropy is often used to assess the classification accuracy of the latent profiles. An entropy value of 0.80 or above typically indicates classification accuracy exceeding 90% (Qiu, 2008; J.-T. Zhang et al., 2010). In addition, to ensure the validity of the model, it is generally recommended that the smallest class account for at least 10% of the total sample (Nylund et al., 2007).

To further explore differences between each profile and the overall sample, significance testing was conducted using *p*-values to determine whether the measured indicators differed significantly from the overall means. Multinomial logistic regression was then employed to examine the influence of demographic variables on latent profiles using the manual three-step approach.

Data analysis was conducted using SPSS 22.0, R Studio 4.4.2, and Mplus 8.0.

### 2.4. Common Method Bias

Because all data in this study were collected through self-report questionnaires, the potential for common method bias was examined to ensure the validity of the findings. Following the procedural recommendations (H. Zhou & Long, 2004), three strategies were implemented to control for common method bias: (1) scientifically optimizing the design of the measurement instruments, (2) standardizing the administration procedure, and (3) including several reverse-coded items.

To statistically assess the presence of common method bias, Harman’s single-factor test was conducted using the 17 measured variables. Results indicated that the first unrotated factor accounted for 16.63% of the variance, which is well below the critical threshold of 40%. This suggests that common method bias was not a significant concern in this study.

## 3. Results

### 3.1. Correlational Analysis Among Variables

Table 1 presents the correlation results among the 17 study variables. The analysis revealed that prosocial behavior was significantly associated with most variables. Specifically, it showed positive correlations with basic psychological needs (autonomy, competence, and relatedness), interpersonal reactivity dimensions (perspective-taking, fantasy, and empathic concern), communal orientation, and prosocial self-efficacy. In contrast, prosocial behavior was negatively correlated with negative life events, including interpersonal stress, adaptation difficulties, academic pressure, and experiences of loss.

Aggressive behavior, on the other hand, exhibited an opposite pattern of associations. It was negatively correlated with all dimensions of basic psychological needs, interpersonal reactivity (perspective-taking, fantasy, empathic concern, and personal distress), communal orientation, and prosocial self-efficacy. Conversely, aggressive behavior showed positive correlations with certain negative life events, specifically interpersonal stress, adaptation difficulties, and experiences of loss.

These findings quantitatively demonstrate the differentiated patterns of associations that prosocial and aggressive behaviors exhibit with key predictive variables.

### 3.2. Latent Profile Model of Prosocial and Aggressive Behaviors

The study constructed behavior tendency classification models with 1 to 6 latent classes and compared six model fit indices to determine the optimal number of categories. Detailed results are presented in Table 2. As shown, when the number of classes reached five or six, the smallest class proportion fell below 10%, which could undermine model stability. Among the models with one to four classes, both AIC and BIC values showed a decreasing trend, and reached their minimum when the number of classes was four. At this point, the ABIC was also minimized, and the entropy value remained at a relatively high level. Therefore, the four-class model was prioritized.

**Figure 1:**
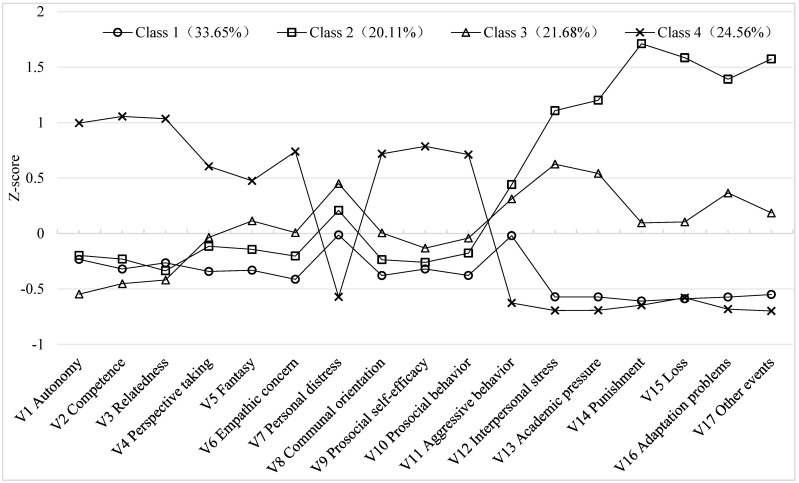
Estimated Conditional Means of Four Profiles on 17 Related Variables (*N* = 1596).

Furthermore, with the four-class model retained, the significance level of classification error was relatively high (*p* < 0.01). Taking all factors into consideration, the four-class model was ultimately selected as the best-fitting solution, as it most accurately captured the characteristics of the study sample. The profile characteristics of each latent class in the four-class model are illustrated in Figure 1.

The classification probabilities for each latent class are presented in Table 3. This matrix displays the average probabilities of assignment from each latent group (rows) to each latent class (columns), ranging from 92.30% to 97.00%. These results indicate high reliability in classification, suggesting that the four-class model has good stability in categorizing participants’ behavioral tendencies.

**Figure 2:**
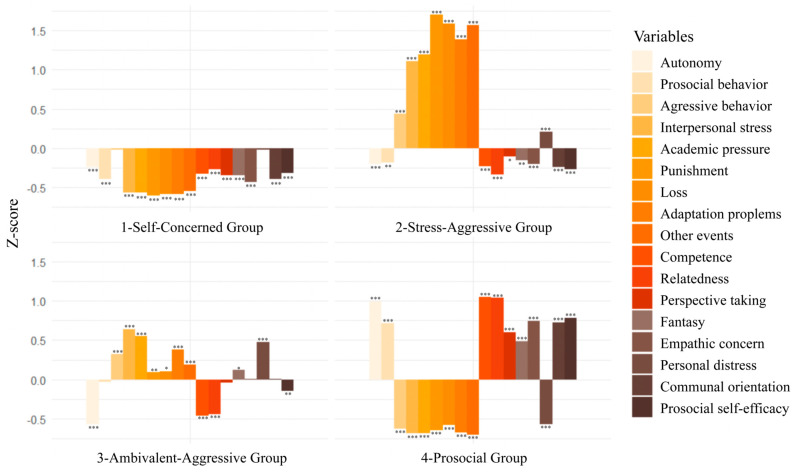
Significance of Variables across Four Profiles (*N* = 1596). Notes: *** *p* < 0.001, ** *p* < 0.01, * *p* < 0.05.

A difference test was conducted for each variable across the profiles in comparison to the overall sample mean. The results, shown in Figure 2, reveal distinct differences across the four latent profiles. Each bar chart depicts how the mean of each measurement indicator deviates from the sample mean (i.e., 0).

In Class C1, all indicators were significantly lower than the sample average (*p* < 0.001), except for personal distress (*p* = 0.62) and aggressive behavior (*p* = 0.63). This suggests that individuals in this group experienced fewer negative life events, lower overall stress, and did not perceive significant anxiety or discomfort. Their basic psychological needs were unmet, empathy levels were low, and they tended to prioritize self-interest. Additionally, they reported low perceived efficacy in engaging in prosocial behaviors. These characteristics reflect a relatively low-engagement and self-oriented profile, and this class was labeled the Self-Concerned Group, accounting for 33.65% of the sample.

In Class C2, all measurement indicators showed statistically significant differences (*p* < 0.001). Participants in this group reported a high number of negative life events and experienced elevated levels of stress, along with notable anxiety and discomfort. Their basic psychological needs were poorly met, perceived prosocial self-efficacy was low, and empathy was also low, with a tendency to prioritize self-interest. This profile reflects a “stress-driven and aggression-compensated” pattern, and was labeled the Stress-Aggressive Group, comprising 20.11% of the sample.

In Class C3, most variables significantly differed from the sample mean, except for perspective taking (*p* = 0.41), empathic concern (*p* = 0.84), communal orientation (*p* = 0.91), and prosocial tendency (*p* = 0.47). Participants in this group scored significantly higher on aggressive tendency, negative life events, fantasy, personal distress, and prosocial self-efficacy (*p* < 0.05), while all dimensions of basic psychological needs were significantly lower than the sample mean (*p* < 0.001). These individuals encountered high levels of stress and unmet psychological needs, exhibited strong aggression, and experienced anxiety and discomfort. Despite showing some empathic traits, their prosocial self-efficacy remained low, reflecting a “need-deprived and ambivalently empathic” pattern. This profile was labeled the Ambivalent-Aggressive Group, accounting for 21.68% of the sample.

In Class C4, all variables differed significantly from the sample mean (*p* < 0.001). This group reported fewer negative life events and lower stress levels, experienced less anxiety and discomfort, and showed higher satisfaction of basic psychological needs. Participants exhibited high levels of empathy, concern for others’ interests, and perceived prosocial self-efficacy. This reflects a “resource-rich and prosocial” profile, and was labeled the Prosocial Group, comprising 24.56% of the sample.

A difference test was conducted on prosocial and aggressive behavior scores for each profile. The results showed that in Class C1, prosocial behavior was significantly lower than aggressive behavior [*t*_(536)_ = −6.87, *p* < 0.001]; in Class C2, prosocial behavior was significantly lower than aggressive behavior [*t*_(320)_ = −7.12, *p* < 0.001]; in Class C3, prosocial behavior was significantly lower than aggressive behavior [*t*_(345)_ = −4.99, *p* < 0.001]; and in Class C4, prosocial behavior was significantly higher than aggressive behavior [*t*_(391)_ = 20.40, *p* < 0.001].

### 3.3. The Influence of Demographic Variables on Latent Profiles

A multinomial logistic regression analysis was conducted to examine the predictive effects of demographic variables on the latent class groupings. Taking the four-category latent profiles as the dependent variable, gender, grade level, father’s education level, mother’s education level, and left-behind experience were entered as independent variables, with Class C1 used as the reference group. The results indicated that gender, grade level, and parental education levels did not have significant predictive power (*p* > 0.05). The overall predictive effect of left-behind experience was significant (*p* < 0.05); however, in the comparison between Class C1 and Class C4, the odds ratio (OR) was 0.84, with a 95% confidence interval of [0.49, 1.19], and this comparison did not reach statistical significance.

## 4. Discussion

Based on Self-Determination Theory, this study adopted a person-centered latent profile analysis approach to explore behavioral differentiation following negative experiences. By examining variables such as empathy, communal orientation, and prosocial self-efficacy, it was found that individuals experiencing different types of negative life events exhibited distinct patterns of aggressive and prosocial behaviors. The results revealed four latent profiles with the following characteristics:

Class C1 demonstrated characteristics of being “self-oriented and amotivated,” exhibiting the lowest level of prosocial behavior among all groups. Despite experiencing relatively low overall life stress, this profile does not reflect a pattern of withdrawal under external pressure, but rather a state of unmet social–psychological needs. Low levels of empathy and prosocial motivation suggest that individuals in this group may avoid or lack the willingness for positive social interactions, which could account for their indifferent attitude toward prosocial behavior following negative life events (Gagné, 2003; Lockwood et al., 2014). From the perspective of Self-Determination Theory, this group may be in an “amotivation” state: lacking both intrinsic motivation (e.g., value-driven desire to help) and extrinsic motivation (e.g., social rewards associated with helping behavior) (Deci & Ryan, 2000). Such a low-motivation, low-behavioral tendency pattern has been partially identified in prior studies as a “detached type” or ‘low sociality’ group (Kanacri et al., 2012). Furthermore, the aggression level in this group did not significantly differ from the overall average, suggesting that their lack of motivation manifests more as behavioral absence rather than deviant behavior, which aligns with prior findings of low-motivation but also low-aggression profiles (Q. Zhou et al., 2002). Over time, although these individuals may not currently be significantly affected by negative life events, their limited motivational and social reserves may hinder their ability to positively cope with future stressors.

Class C2 displayed a “stress-driven and aggression-compensating” pattern, consistent with existing research suggesting that the accumulation of negative life events may weaken individuals’ motivation for social connection and belief in their helping capabilities, thus intensifying aggressive behavioral responses (Brown et al., 2017). The group’s empathy, communal orientation, and prosocial self-efficacy were all lower than the sample average, indicating a tendency to focus on the self rather than others and a lack of belief in their capacity to help. Under high-stress conditions, these individuals are more likely to adopt externalizing behaviors such as aggression as a compensatory psychological response. This finding aligns with previous studies suggesting that individuals chronically exposed to stressors and lacking concern for others are more prone to vent through aggressive behavior (Martin-Key, 2017; Rutter & Atkinson, 2023). Compared to the C1 group (the “self-focused” profile) both groups shared low levels of basic psychological need satisfaction. However, C2 reported significantly higher stress intensity and emotional distress, which may explain their elevated levels of aggression. Notably, within this profile, punishment and loss appeared to be elevated to a slightly greater extent relative to the sample mean than adaptation problems, interpersonal stress, and academic pressure. This pattern suggests that experiences involving punishment and loss may be particularly salient sources of stress for individuals in this group, potentially contributing to their stronger externalizing responses under pressure. This contrast further underscores the potential interaction between high stress and motivational deficiency in amplifying aggressive tendencies. Accounting for 20.11% of the sample, this group highlights the existence of a “high stress–low motivation–high aggression” subgroup within the college student population. Therefore, psychological interventions for this group should target both stress alleviation and enhancement of emotional regulation and prosocial motivation training to effectively improve behavioral outcomes.

Class C3 demonstrated a “need-deprived and ambivalent empathy” profile. This group’s scores on empathic concern and perspective-taking did not significantly differ from the overall mean, but their scores on fantasy and personal distress were significantly higher. This may suggest that while these individuals possess certain emotional sensitivity and responsiveness, they fail to establish an effective connection between prosocial motivation and behavior. Previous studies have noted that empathy is not inherently protective; aggressive behavior may be influenced by multiple moderating variables such as emotional regulation ability, value orientation, and situational stress (Roberton et al., 2011). Furthermore, Decety and Cowell (2014) proposed that under conditions of strong in-group vs. out-group differentiation, empathy can become “selectively biased”, where individuals show high empathy toward in-group members but may still exhibit aggression toward out-group individuals. This perspective may help explain why certain empathy dimensions were elevated in this group, while their aggression level remained high—empathy experiences may not translate into tolerance or support under specific social situations (e.g., perceived injustice or isolation), but instead may trigger emotional conflict and hostility. Additionally, this group’s significantly elevated “personal distress” suggests that they are more likely to experience self-focused discomfort in response to others’ suffering, rather than engaging in empathy driven by prosocial motivation. Notably, the results also showed that interpersonal and academic stress were elevated to a relatively greater extent above the sample mean compared with punishment and loss, suggesting that social and performance-related stressors may represent particularly salient challenges for this group. Such stress patterns are consistent with their emotional ambivalence and heightened reactivity, potentially amplifying both empathic distress and aggressive tendencies under unmet psychological needs. This type of self-centered emotional involvement is often positively associated with aggression (Roberton et al., 2011) and may reflect a state of “moral fatigue,” in which prolonged exposure to high stress and unmet basic needs erodes one’s capacity to sustain moral behaviors (Muraven & Baumeister, 2000). Accounting for 21.68% of the total sample, this group points to the existence of a unique subgroup characterized by the coexistence of empathy and aggression. These individuals are not incapable of perceiving others’ emotions, but due to high-stress conditions, low self-efficacy, and insufficient emotional regulation, empathy fails to function as a protective factor. This finding suggests that interventions should go beyond simply ‘enhancing empathy’ and instead address the imbalance between motivation, capacity, and external context.

Class C4 exhibited a “resource-rich and prosocial” profile, consistent with prior research on prosocial personality traits and behavioral tendencies. Existing studies indicate that individuals with high empathy and strong prosocial beliefs are more likely to offer help spontaneously when others are in need (Eisenberg et al., 2006). These individuals often possess effective emotion regulation skills, strong intrinsic motivation, and a sense of social belonging (Caprara et al., 2005). Moreover, the high level of basic psychological needs satisfaction in this group suggests that they enjoy robust social support, contextual resources, and self-efficacy, all of which are recognized as key predictors of prosocial behavior (Deci & Ryan, 2000; Pavey et al., 2011). Within the present sample, this group displayed the most positive overall psychological state, indicating that when motivational systems (e.g., need satisfaction) and resource systems (e.g., empathy, low stress) are well-coordinated, individuals are more likely to exhibit stable prosocial tendencies. This also suggests that future intervention designs should focus on activating prosocial potential through multiple pathways, such as enhancing social support, fostering self-efficacy, and improving emotional regulation.

We also examined whether demographic variables (e.g., gender, grade level, parental education, and left-behind experience) could predict membership in the identified latent profiles. The results indicated no significant predictive effects, suggesting that these basic background characteristics may play a limited role in shaping the behavioral and motivational profiles. This finding highlights the potential importance of psychological and contextual factors in explaining individual differences and provides directions for future research.

Although this study did not further differentiate sub-types of aggressive and prosocial behavior, and the sample age range was relatively narrow, the latent profile analysis nonetheless revealed preliminary classifications of individuals based on negative life events, motivational processes, and behavioral tendencies. The findings suggest that individuals are more likely to exhibit positive behavioral tendencies when autonomy, empathy, and self-efficacy are well aligned, providing insight into the formation of prosocial behavior following adversity. Future studies could further validate the stability of these classifications across broader age groups and employ longitudinal designs to examine the dynamic evolution between negative experiences and prosocial behavior.

## 5. Conclusions

The results of this study indicate that aggressive behavior is associated with a higher frequency of negative life experiences, while prosocial behavior is linked to fewer such experiences. The two behavioral tendencies follow different motivational transformation pathways, with empathy, particularly the personal distress dimension, playing a key role in both. The findings also reveal heterogeneity within subgroups of aggressive behavior and confirm significant positive associations between prosocial behavior, empathy, communal orientation, and prosocial self-efficacy. These results, from both emotional–motivational and social–cognitive perspectives, provide empirical support for understanding behavioral differentiation mechanisms and for developing corresponding intervention strategies.

## Figures and Tables

**Table 1:** Correlations Among Measured Variables.

	1	2	3	4	5	6	7	8	9	10	11	12	13	14	15	16	17
1. Autonomy	1																
2. Competence	0.75 ***	1															
3. Relatedness	0.73 ***	0.71 ***	1														
4. Perspective taking	0.23 ***	0.25 ***	0.30 ***	1													
5. Fantasy	0.13 ***	0.21 ***	0.21 ***	0.38 ***	1												
6. Empathic concern	0.25 ***	0.29 ***	0.35 ***	0.38 ***	0.40 ***	1											
7. Personal distress	−0.43 ***	−0.43 ***	−0.31 ***	−0.02	0.08**	−0.01	1										
8. Communal orientation	0.24 ***	0.32 ***	0.38 ***	0.37 ***	0.41 ***	0.57 ***	0.01	1									
9. Prosocial self-efficacy	0.38 ***	0.43 ***	0.46 ***	0.41 ***	0.27 ***	0.40 ***	−0.15 ***	0.46 ***	1								
10. Prosocial behavior	0.25 ***	0.29 ***	0.33 ***	0.45 ***	0.27 ***	0.45 ***	0.02	0.49 ***	0.50 ***	1							
11. Aggressive behavior	−0.31 ***	−0.29 ***	−0.33 ***	−0.19 ***	−0.05 *	−0.21 ***	0.30 ***	−0.17 ***	−0.28 ***	−0.21 ***	1						
12. Interpersonal stress	−0.33 ***	−0.28 ***	−0.32 ***	−0.05	0.06 *	−0.07 **	0.31 ***	−0.03	−0.16 ***	−0.08 ***	0.36 ***	1					
13. Academic pressure	−0.34 ***	−0.26 ***	−0.29 ***	−0.03	0.03	−0.07 **	0.26 ***	−0.03	−0.11 ***	−0.08 ***	0.30 ***	0.77 ***	1				
14. Punishment	−0.17 ***	−0.18 ***	−0.22 ***	−0.06 *	−0.05	−0.11 ***	0.17 ***	−0.12 ***	−0.14 ***	−0.10 ***	0.27 ***	0.69 ***	0.76 ***	1			
15. Loss	−0.13 ***	−0.14 ***	−0.19 ***	−0.07 **	−0.04	−0.11 ***	0.11 ***	−0.11 ***	−0.12 ***	−0.09 ***	0.23 ***	0.61 ***	0.66 ***	0.82 ***	1		
16. Adaptation problems	−0.27 ***	−0.24 ***	−0.27 ***	−0.04	0.02	−0.08 **	0.23 ***	−0.05	−0.15 ***	−0.07 **	0.31 ***	0.72 ***	0.77 ***	0.75 ***	0.73 ***	1	
17. Other events	−0.25 ***	−0.24 ***	−0.27 ***	−0.08 **	−0.01	−0.12 ***	0.23 ***	−0.12 ***	−0.19 ***	−0.12 ***	0.35 ***	0.70 ***	0.70 ***	0.85 ***	0.73 ***	0.78 ***	1
*M*	4.65	4.67	5.17	3.67	3.52	3.61	3.11	3.44	5.58	3.63	2.08	1.53	1.46	0.84	1.07	1.29	0.95
*SD*	0.84	0.93	0.89	0.59	0.58	0.54	0.74	0.39	0.90	0.53	0.72	1.09	1.00	1.11	1.37	0.97	0.99

Notes: *N* = 1596, *** *p* < 0.001, ** *p* < 0.01, * *p* < 0.05.

**Table 2:** Fit Indices for 1–6 Classes in Latent Profile Analysis.

Class	AIC	BIC	ABIC	LMRT	BLR	Entropy	Minimum Classification Proportion
1	77,048.28	77,231.03	77,123.02	/	/	/	
2	70,107.21	70,386.72	70,221.53	0.000	0.000	0.964	27.19%
3	67,297.66	67,673.92	67,451.55	0.030	0.000	0.904	24.12%
**4**	**65,878.16**	**66,351.18**	**66,071.62**	**0.006**	**0.000**	**0.887**	**20.11%**
5	64,848.62	65,418.40	65,081.66	0.064	0.000	0.891	9.21%
6	64,162.19	64,828.72	64,434.80	0.095	0.000	0.893	9.15%

Notes: Bold values indicate the fit indices used to determine the final four-class solution.

**Table 3:** Average Assignment Probabilities (Columns) of Participants (Rows) in Each Latent Class.

	C-1(%)	C-2(%)	C-3(%)	C-4(%)
C1	92.30	0.00	3.73	3.93
C2	0.00	97.00	3.00	0.03
C3	5.18	1.52	92.80	0.48
C4	6.26	0.00	1.47	92.30

## Data Availability

The data presented in this study are available on request from the corresponding author due to privacy and ethical restrictions.
